# Echocardiographic strain as an ally in the evaluation of congenital heart disease: a narrative review

**DOI:** 10.3389/fcvm.2026.1662430

**Published:** 2026-04-22

**Authors:** Yesika Lucero Alvarez Alarcón, Lina Paola Montaña-Jimenez

**Affiliations:** 1 Research Seedbed, Pontificia Universidad Javeriana, Bogotá, Colombia; 2 Department of Pediatrics, Pontificia Universidad Javeriana, Bogotá, Colombia; 3 Division of Pediatric Cardiology, Clínica del Country, HOMI Fundación Hospital Pediátrico de la Misericordia, Bogotá, Colombia

**Keywords:** congenital heart disease, fetal echocardiography, myocardial deformation, myocardial strain, pediatric cardiology, speckle-tracking echocardiography

## Abstract

**Introduction:**

Congenital heart diseases (CHDs) are the most common congenital malformations in newborns and are associated with significant long-term cardiac morbidity. Conventional echocardiographic parameters such as ejection fraction often fail to detect subclinical myocardial dysfunction. Myocardial strain analysis, primarily through speckle-tracking echocardiography (STE), has emerged as a sensitive, non-invasive technique to assess myocardial deformation and guide clinical decision-making in pediatric patients with CHDs.

**Methods:**

This narrative review was conducted through a comprehensive search of PubMed, Embase, and Scopus databases, covering literature from 2009 to 2025. Search terms included “global longitudinal strain,” “congenital heart disease,” and pediatric age-related keywords. A total of 119 records were screened, and 40 studies were selected based on relevance and methodological quality. Articles were included regardless of language but ultimately analyzed in English or Spanish.

**Results:**

The reviewed studies confirm the clinical utility of strain imaging in detecting early myocardial dysfunction, monitoring surgical outcomes, and predicting prognosis across various CHDs, including atrial and ventricular septal defects, Tetralogy of Fallot, single-ventricle physiology, and transposition of the great arteries. RV longitudinal strain and atrial strain have shown prognostic value in early dysfunction and adverse outcomes. Additionally, fetal myocardial strain imaging has demonstrated utility in prenatal diagnosis and individualized care planning.

**Conclusion:**

Myocardial strain imaging is a powerful adjunct to conventional echocardiography, offering enhanced sensitivity for identifying subclinical myocardial dysfunction. Its integration into routine practice enhances risk stratification, informs therapeutic decisions, and contributes to personalized care in CHD across all stages of life, including the fetal period. Its systematic use is strongly recommended in pediatric cardiology centers specializing in CHD.

## 1 Introduction

CHDs represent the most common congenital malformation in live-born infants, with an estimated incidence of approximately 7.2 per 1,000 births in developing countries. These conditions encompass a broad spectrum of anatomical and functional cardiac abnormalities that can significantly impact both quality of life and life expectancy if not diagnosed and managed promptly (1). In the pediatric setting, where clinical presentation may be subtle or even asymptomatic during the early stages, accurate, accessible, and non-invasive diagnostic tools are essential for proper structural and functional assessment of the cardiovascular system.

Echocardiography has become the most widely used imaging modality for cardiac evaluation in children, both in hospital and outpatient settings. Its ability to provide real-time information on cardiac anatomy, systolic and diastolic function, and valvular dynamics makes it an indispensable tool (2, 3). However, the interpretation of conventional parameters, such as ejection fraction (EF), may be insufficient to detect early myocardial abnormalities—particularly in patients with a preserved EF but ongoing symptoms or unfavorable clinical progression. In this context, myocardial deformation analysis, or strain, has emerged as an advanced technique capable of evaluating myocardial mechanical function with greater sensitivity and specificity (4).

Myocardial strain quantifies the fractional change in myocardial fiber length throughout the cardiac cycle and serves as a sensitive marker of myocardial deformation and systolic performance at both regional and global levels (5). Unlike ejection fraction, which is derived from volumetric assumptions and is strongly influenced by ventricular geometry, strain analysis allows the detection of subtle myocardial dysfunction even when conventional parameters remain within normal ranges. Although strain is less dependent on geometric assumptions than ejection fraction, it remains influenced by loading conditions, a consideration that is particularly relevant in CHD, where abnormal anatomy and altered loading frequently complicate functional assessment using traditional indices (6).

**Figure 1:**
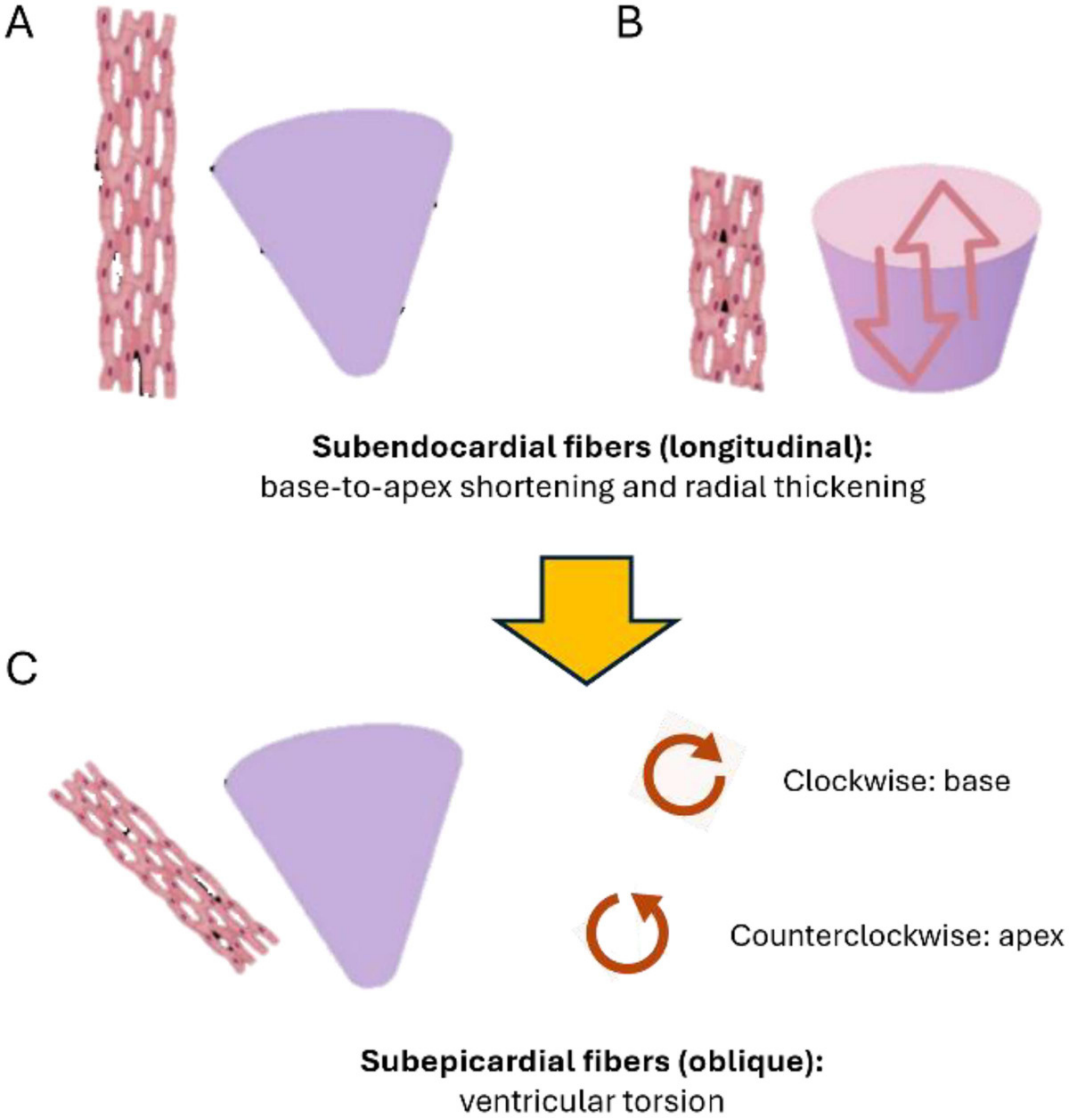
Myocardial deformation in different planes. Illustration of myocardial fiber orientation and the resulting deformation mechanics. **(A,B)** Longitudinal strain is generated by subendocardial fibers, producing base-to-apex shortening and radial thickening. Circumferential strain results from mid-wall fibers contracting around the ventricular circumference. **(C)** Oblique subepicardial fibers induce ventricular torsion, characterized by clockwise basal and counterclockwise apical rotation when viewed from the apex. These three components—longitudinal, circumferential, and torsional deformation—act synergistically to optimize systolic ejection. Image adapted with permission from Dr. Lina Montaña.

The most widely validated technique for measuring myocardial deformation in the pediatric population is speckle-tracking echocardiography (STE), which relies on acoustic marker tracking. This modality is highly advantageous as it is angle-independent and allows for a comprehensive assessment of multiple spatial planes. As illustrated in Figure 1, STE facilitates the evaluation of longitudinal, circumferential, and radial mechanics, demonstrating high reproducibility under optimal imaging conditions. Its clinical application has steadily expanded to a variety of congenital lesions, including ventricular septal defect, atrial septal defect, tetralogy of Fallot, transposition of the great arteries, and single-ventricle physiology. In these settings, strain imaging serves as a valuable adjunctive tool for detecting subclinical ventricular dysfunction, monitoring the response to surgical interventions, and assisting in the formulation of short- and long-term prognoses (5).

Similarly, strain analysis of the Right Ventricle (RV) has gained increasing relevance, as this chamber is frequently involved in various CHDs—either as the systemic ventricle or in settings of chronic pressure or volume overload (7). In such cases, the RV may undergo compensatory structural adaptations, such as concentric or eccentric hypertrophy, which initially preserve cardiac output but eventually progress to dysfunction. Strain imaging enables more precise characterization of these evolutionary stages, facilitating earlier and more targeted medical or surgical interventions (8).

Therefore, strain analysis in children with CHD not only enhances the early detection of myocardial dysfunction but has also become a valuable tool for guiding therapeutic decisions, optimizing clinical follow-up, and improving postoperative outcomes (9).

While previous guidelines and expert consensus documents have established the foundational physics of myocardial deformation and its prognostic value in adult cardiology, a significant gap remains regarding a critical, lesion-specific synthesis dedicated exclusively to the pediatric and fetal spectrum. Prior narrative reviews have frequently presented strain as a purely descriptive metric or focused on isolated congenital defects. This review differentiates itself by bridging the gap between evolving technical guidelines and practical, day-to-day clinical implementation across a broad range of congenital heart diseases (CHD). Furthermore, given the recent technological evolution in speckle-tracking algorithms—including improved temporal resolution for fetal assessment and the ongoing transition toward vendor-neutral platforms—an updated, expert-guided synthesis is highly timely. Therefore, this narrative review aims to critically evaluate the current strength of evidence, highlight the nuanced boundaries between load-dependent deformation and true contractility, and provide a balanced perspective on the clinical adoption of strain imaging in pediatric cardiology.

## 2 Literature search strategy

To construct this narrative review, a comprehensive literature search was conducted across major electronic databases, including PubMed, Scopus, and Embase, focusing on literature published up to 2025. The search incorporated terms related to STE, myocardial strain, fetal cardiology, and pediatric CHD. Rather than employing a rigid systematic extraction protocol, articles were purposefully selected based on their clinical relevance, historical significance, methodological quality, and contribution to the evolving understanding of ventricular mechanics. This approach allowed for a broad and critical synthesis of current evidence, observational studies, and technical limitations, aligning with the educational and clinical scope of a narrative review.

## 3 Cardiac structure and function

The assessment of biventricular mechanics must account for the distinct architectural and functional properties of each chamber. In the left ventricle (LV), the complex arrangement of myocardial fibers facilitates a coordinated contraction that propagates from the endocardium to the epicardium and from apex to base (10, 11). While traditional imaging often reduces LV performance to simple volumetric ejection fractions, this multidirectional deformation is essential to generate adequate systemic pressures. Consequently, advanced techniques like STE are increasingly necessary to capture these subtle mechanical changes before gross volumetric declines occur (12).

The RV presents a uniquely complex imaging challenge. Anatomically, its thin-walled, highly trabeculated, crescentic structure renders precise endocardial delineation and the geometric assumptions required by conventional 2D echocardiography inherently flawed (12). Physiologically, RV systolic performance is predominantly driven by the longitudinal shortening of its subendocardial fibers, with secondary contributions from inward free-wall movement and septal displacement (12). Because longitudinal deformation is the primary driver of RV ejection, RV global longitudinal strain (RV-GLS) emerges as a superior, geometry-independent metric for assessing RV function, particularly in congenital scenarios characterized by altered loading conditions or complex ventricular interdependence.

Similarly, atrial evaluation extends beyond mere anatomical or volumetric measurements. The atria play a critical, dynamic role in modulating ventricular filling through three distinct phases: acting as a reservoir during ventricular systole, a passive conduit during early diastole, and an active pump in late diastole (10, 13). In CHD, where ventricular compliance is often compromised, evaluating these phasic functions via atrial strain provides early diagnostic markers of elevated filling pressures and diastolic dysfunction, long before overt atrial dilation becomes evident on standard imaging (14).

### 3.1 Heart failure

Heart failure, as defined by the American Heart Association (AHA) in 2022, is a clinical syndrome characterized by typical signs and symptoms attributable to a structural and/or functional cardiac abnormality that leads to reduced cardiac output and/or elevated intracardiac pressures, either at rest or during exertion (15).

Among its phenotypes, heart failure with preserved ejection fraction (HFpEF) is characterized by a condition in which the heart maintains an EF within the normal range but exhibits impaired ventricular filling. This dysfunction is associated with reduced ventricular compliance and relaxation capacity, which hinders adequate diastolic filling and leads to congestion and clinical symptoms despite preserved systolic function (15).

The diagnostic evaluation of HFpEF includes tools such as echocardiography and cardiac catheterization, with the former being the most widely used in clinical practice. Relevant echocardiographic findings include RV size, assessed through end-diastolic diameter and its ratio to the LV; RV wall thickness, which helps detect hypertrophy secondary to pressure overload; and parameters of RV systolic function such as TAPSE (Tricuspid Annular Plane Systolic Excursion) and the myocardial performance index (or Tei index), which integrates isovolumetric contraction and relaxation times relative to ejection time (16).

Additionally, indirect hemodynamic indicators are assessed, including pulmonary artery systolic pressure (PASP), estimated from tricuspid regurgitation velocity, and proper atrial pressure, inferred from the diameter and collapsibility of the inferior vena cava (16).

Myocardial strain is a dimensionless measurement that describes myocardial deformation, providing additional information about cardiac function even when LV ejection fraction (LVEF) is preserved. This is because radial and circumferential functions may compensate for subclinical longitudinal dysfunction, allowing the LVEF to remain within normal limits (6).

RV strain should be interpreted in the context of factors such as preload, afterload, and chamber geometry. In strain analysis, the evaluation typically focuses on the RV-free wall, where average longitudinal deformation is approximately 30% and circumferential strain is around 15%. This contrasts with the LV, in which average longitudinal and circumferential strain values are approximately 20% and 25%, respectively (12).

RV longitudinal strain has proven to be a sensitive marker for detecting subclinical dysfunction and predicting prognosis in conditions involving pressure or volume overload, such as pulmonary hypertension, severe tricuspid regurgitation, or infections like COVID-19 (12). In states of RV hypertrophy, a reduction in longitudinal strain is commonly observed, accompanied by an increase in the circumferential component. Additionally, the presence of intraventricular dyssynchrony can lead to a heterogeneous distribution of wall stress, accelerating RV functional deterioration (12).

A diastolic leftward shift of the interventricular septum can induce simultaneous diastolic dysfunction of the LV (12). In many conditions, ventricular strain remains reduced even after surgical correction, suggesting the presence of incomplete reverse remodeling or irreversible myocardial damage (12).

On the other hand, left atrial (LA) function and structural quantification are key aspects in detecting subclinical atrial disease. LA dysfunction has been associated with the development of heart failure, particularly with preserved ejection fraction, due to its close relationship with LV diastolic function (3). Moreover, in patients with CHD, early LA dysfunction is linked to an increased cardiovascular risk, highlighting the importance of early assessment as a preventive measure against the development of heart failure symptoms (3).

## 4 Speckle-tracking echocardiographic strain

Echocardiography is the first-line modality for evaluating ventricular function. However, due to the heart's complex and variable anatomy, acquiring accurate images can be challenging and highly dependent on the operator's experience. Other limitations include suboptimal acoustic windows, inaccurate geometric assumptions, and the presence of postoperative sternal deformities or a robust body habitus, all of which can compromise image quality (2, 3).

Myocardial deformation imaging has emerged as a valuable tool for assessing cardiac function. It can be obtained using tissue Doppler imaging; however, the most widely used technique today is STE, which is angle-independent and unaffected by the translational motion of the heart (12, 17–19). This non-invasive modality enables quantification of both regional and global myocardial deformation throughout the cardiac cycle, represented as a percentage change. It is more reproducible and feasible than Doppler-based methods (1, 12, 18).

### 4.1 Myocardial deformation parameters

**Figure 2:**
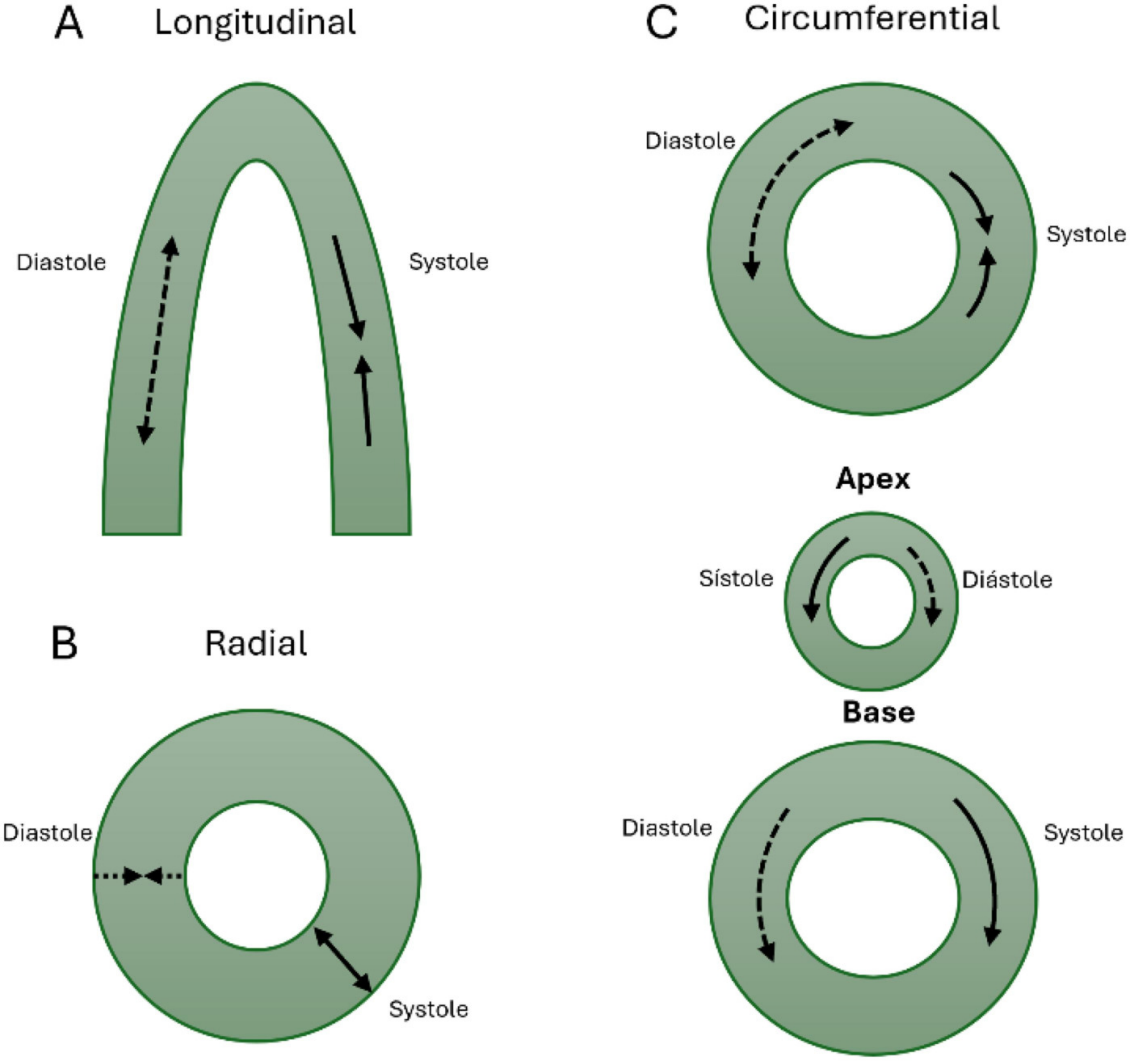
Types of myocardial deformation and torsional mechanics. Illustration of the main components of myocardial deformation: **(A)** longitudinal deformation, **(B)** radial deformation (wall thickening), and **(C)** circumferential deformation (contraction around the ventricular circumference). Apical and basal rotation occur in opposite directions, resulting in ventricular torsion.

Speckle-tracking echocardiography allows for a comprehensive, multidirectional assessment of ventricular mechanics. As conceptually illustrated in Figure 2, myocardial deformation is quantified across three primary spatial planes (18). Longitudinal strain measures the systolic shortening of myocardial fibers along the long axis (expressed as a negative value) and is widely utilized for both segmental and global ventricular assessment. Conversely, radial strain reflects myocardial thickening directed toward the center of the chamber, while circumferential strain represents the shortening of fibers along the circular perimeter (18). Furthermore, the temporal derivative of these variables, known as the strain rate, describes the speed of deformation over time. Although strain rate is theoretically less volume-dependent, its clinical application in pediatric and fetal populations is often constrained by the necessity for exceptionally high temporal resolution to accommodate rapid heart rates (18).

In adults, a global longitudinal strain (GLS) value of greater than 20% (absolute) is considered normal for the RV (18, 20). However, robust normative data indicate that normal right ventricular deformation in pediatric populations is often of greater magnitude than in adults, with healthy children demonstrating mean RV-GLS values around −24.4% and RV free-wall strain (RV-FWS) values approaching −27.2% (21). These values may also vary depending on the specific software used for analysis (e.g., GE, Philips, Siemens, Esaote) (18, 20).

Regarding reference ranges for left ventricular strain measurements, values reported in the pediatric population are derived from systematic reviews and meta-analyses encompassing a wide age spectrum, from infancy to late adolescence (22). These ranges should not be interpreted as indicating direct equivalence of strain values across all pediatric ages. Myocardial strain parameters exhibit age-related variations, particularly during early childhood, reflecting developmental changes in myocardial structure, ventricular geometry, heart rate, and loading conditions.

The interpretation of myocardial strain in pediatric populations necessitates a nuanced approach, as deformation parameters exhibit significant age-dependent maturation. Specifically, left ventricular global longitudinal strain (LV-GLS) tends to be arithmetically lower (less negative) in neonates and infants, progressively increasing toward more stable values by adolescence (22). Consequently, applying a uniform threshold across all age groups is clinically inappropriate. While published normative data suggest that pediatric LV-GLS generally ranges between −16.7% and −23.6% (mean −20.2%), and global circumferential strain shows wider variance (−12.9% to −31.4%), these parameters must be strictly interpreted using age-, body surface area-, and vendor-specific reference values (22). Recent large-cohort pediatric studies emphasize that morphological parameters, particularly body surface area (BSA) and age, significantly correlate with both left and right ventricular strain values. Consequently, the application of standardized Z-score equations is strongly recommended to accurately track maturational changes in clinical practice (21).

Furthermore, it is critical to recognize that speckle-tracking derived strain is not a load-independent metric of true contractility. Myocardial deformation is inherently influenced by hemodynamic loading conditions; alterations in ventricular preload and afterload—which are ubiquitous in congenital heart lesions like significant left-to-right shunts or valvular regurgitation—will directly impact the extent of myocardial shortening (23). Therefore, strain measurements cannot be interpreted in isolation and must always be contextualized within the patient's specific and dynamic hemodynamic state.

### 4.2 Ventricular strain

The assessment of LV strain has become an essential tool for detecting and quantifying subtle alterations in longitudinal systolic function, even in subclinical stages. During systole, the LV undergoes shortening in both the longitudinal and circumferential planes, along with radial thickening. Strain analysis (particularly GLS) is highly sensitive for detecting LV dysfunction, even when EF remains preserved (24).

LV longitudinal strain is considered one of the earliest indicators of ventricular dysfunction. However, it does not directly represent the myocardium's intrinsic contractility, as it is afterload-dependent and subject to some degree of interobserver variability. Subendocardial fibers, which are primarily responsible for longitudinal deformation, are particularly susceptible to ischemia due to their anatomical location and orientation. In this context, longitudinal dysfunction may be compensated by a relative increase in circumferential fiber function, allowing the EF to remain within normal limits despite underlying functional impairment (8).

The evaluation of RV strain—particularly along the longitudinal axis—has been identified as a sensitive and early predictor of RV dysfunction in patients with heart failure. Given the complex geometry of the RV, which limits the accuracy of traditional assessment methods, myocardial deformation analysis has become a key tool for detecting early functional impairments before structural or clinical changes become evident (25).

When evaluating the right ventricle, a critical methodological distinction must be made between RV-GLS (incorporating all six segments, including the interventricular septum) and RV free-wall strain (RV-FWS, limited to the three lateral segments). In the context of CHD, expert consensus increasingly favors RV-FWS (26). The interventricular septum is structurally shared and mechanically interdependent with the LV; furthermore, its motion is frequently paradoxical following cardiac surgery or in the presence of right ventricular volume/pressure overload. Consequently, including the septum in RV strain calculations can either mask isolated RV free-wall dysfunction or falsely lower the overall value due to LV impairment (26). Therefore, RV-FWS is generally considered a more specific indicator of intrinsic right ventricular performance (12, 18, 26).

However, when interpreting these metrics, clinicians must recognize a fundamental physiological caveat: myocardial deformation is not synonymous with true contractility. Strain is inherently load-dependent. In congenital lesions characterized by severe left-to-right shunts (altered preload) or outflow tract obstructions (elevated afterload), abnormal strain values may initially reflect these abnormal loading conditions rather than intrinsic myocardial failure. Therefore, strain metrics should not be interpreted as absolute measures of contractility, but rather as dynamic indicators of myocardial performance that are inextricably linked to the patient's specific hemodynamic state (26).

### 4.3 Atrial strain

Beyond ventricular mechanics, the clinical utility of speckle-tracking echocardiography has expanded to the comprehensive evaluation of atrial deformation. Rather than merely acting as passive transport chambers, the atria possess complex, triphasic mechanics that can be finely quantified using strain imaging. Specifically, left atrial (LA) strain analysis provides a highly sensitive, early marker of left ventricular diastolic dysfunction by evaluating three distinct physiological phases: the reservoir phase (systolic filling), the conduit phase (passive early diastolic transfer), and the booster pump phase (active late diastolic contraction) (3, 27). Because atrial deformation becomes impaired before significant volumetric enlargement occurs, diminished strain and strain rate values serve as early indicators of elevated filling pressures and reduced ventricular compliance.

Similarly, right atrial (RA) strain is increasingly recognized as a critical prognostic indicator, particularly in conditions characterized by altered right heart hemodynamics. In the context of pulmonary hypertension or right-sided heart failure, RA strain tightly correlates with right ventricular stiffness and worsening diastolic function (18). Consequently, integrating biatrial strain into pediatric echocardiographic evaluations offers a non-invasive, highly sensitive window into the diastolic performance of the corresponding ventricles, allowing for earlier risk stratification prior to the onset of overt clinical symptoms.

### 4.4 Limitations

A critical appraisal of the current literature reveals a gap between observational evidence and practical clinical implementation. The majority of studies highlighting the prognostic value of myocardial strain in pediatric CHD remain observational and retrospective (28). While these investigations consistently prove that strain can detect subclinical dysfunction earlier than conventional metrics (such as ejection fraction), there is a distinct lack of large-scale, prospective randomized trials (29). Consequently, the strength of evidence supporting isolated strain-guided clinical decision-making (such as timing for surgical intervention) is not yet robust enough to supersede established multiparametric clinical criteria.

Despite its immense clinical promise, the widespread adoption of myocardial strain imaging in pediatric and fetal cardiology is currently hindered by several significant limitations. A primary, well-documented challenge is inter-vendor variability and proprietary software differences (26, 30). Because speckle-tracking algorithms differ among manufacturers, strain measurements cannot be used interchangeably across different echocardiographic platforms, complicating longitudinal follow-up. Furthermore, strain analysis is heavily dependent on optimal image quality and temporal resolution. In pediatric populations, where resting heart rates are physiologically higher, achieving an adequate frame rate without compromising spatial resolution remains a persistent technical hurdle (31).

Additionally, there is a distinct lack of universally accepted, standardized pediatric and fetal reference ranges, making the interpretation of borderline values challenging (32). Extrapolating adult thresholds to pediatric patients is conceptually flawed, particularly in the context of complex congenital anatomies (33). While this lack of universal Z-scores remains a significant hurdle, recent multicenter efforts utilizing large cohorts of healthy children are beginning to establish reliable, age- and BSA-adjusted Z-score equations for both ventricles, paving the way for more standardized clinical applications (21). In scenarios such as a systemic RV or a single-ventricle (Fontan) circulation, altered loading conditions and atypical ventricular geometry make standard deformation models less reliable (34).

Finally, while automated tracking has improved, assessing the thin-walled pediatric or fetal myocardium remains susceptible to intra- and inter-observer variability. Addressing these issues through standardized protocols and vendor-neutral software is a crucial prerequisite before strain can be routinely integrated into clinical algorithms (28).

## 5 Applications of strain in congenital heart disease

CHDs are the most common isolated fetal malformations, with an estimated incidence of 7.2 per 1,000 live births (1). In these patients, evaluating the response to hemodynamic overload is essential. Unlike acquired heart disease, pressure overload in CHD tends to be better tolerated, highlighting important differences in the clinical course of these conditions (12).

Subjective functional assessment in patients with CHD can be limited, as many present clinically asymptomatic. In this context, objective tools such as strain analysis via echocardiography enable a more accurate evaluation of myocardial function (35). Several studies have shown that myocardial deformation parameters tend to progressively deteriorate from early stages in these patients (36), with RV strain analysis being instrumental as a sensitive marker of early dysfunction (18).

In CHD, the RV is frequently subjected to increased afterload—often more pronounced than in the LV—leading to a more significant reduction in functional parameters and a higher susceptibility to acute RV failure. In chronic pressure overload states, the RV initially responds with adaptive concentric hypertrophy aimed at reducing wall stress. However, once this compensatory mechanism is exhausted, it may progress to eccentric hypertrophy, accompanied by progressive dilation and ventricular dysfunction. In contrast, volume overload conditions also lead predominantly to eccentric hypertrophy, although this type of overload is generally better tolerated than pressure overload (12).

In patients with CHDs such as atrial septal defect (ASD), ventricular septal defect (VSD), tetralogy of Fallot, or AV septal defects corrected by biventricular repair, a reduction in LV longitudinal strain has been documented, even in the presence of preserved ejection fraction. However, some studies have reported a progressive recovery of this parameter during postoperative follow-up. In contrast, circumferential strain may remain elevated, depending on the segment evaluated and the patient's age (37).

In patients with valvular disease, such as severe and critical pulmonary stenosis, longitudinal and segmental strain assess pathological changes in the interventricular septum that are not detected by conventional echocardiographic evaluation. This parameter is one of the key criteria for determining medical or surgical management (38).

In the surgical setting, GLS has been proposed as an early predictor of 30-day morbidity and mortality in patients undergoing cardiac surgery with cardiopulmonary bypass. Preoperative GLS values below −12% have been associated with higher inotropic requirements, prolonged mechanical ventilation, and increased risk of postoperative adverse events (9).

**Figure 3:**
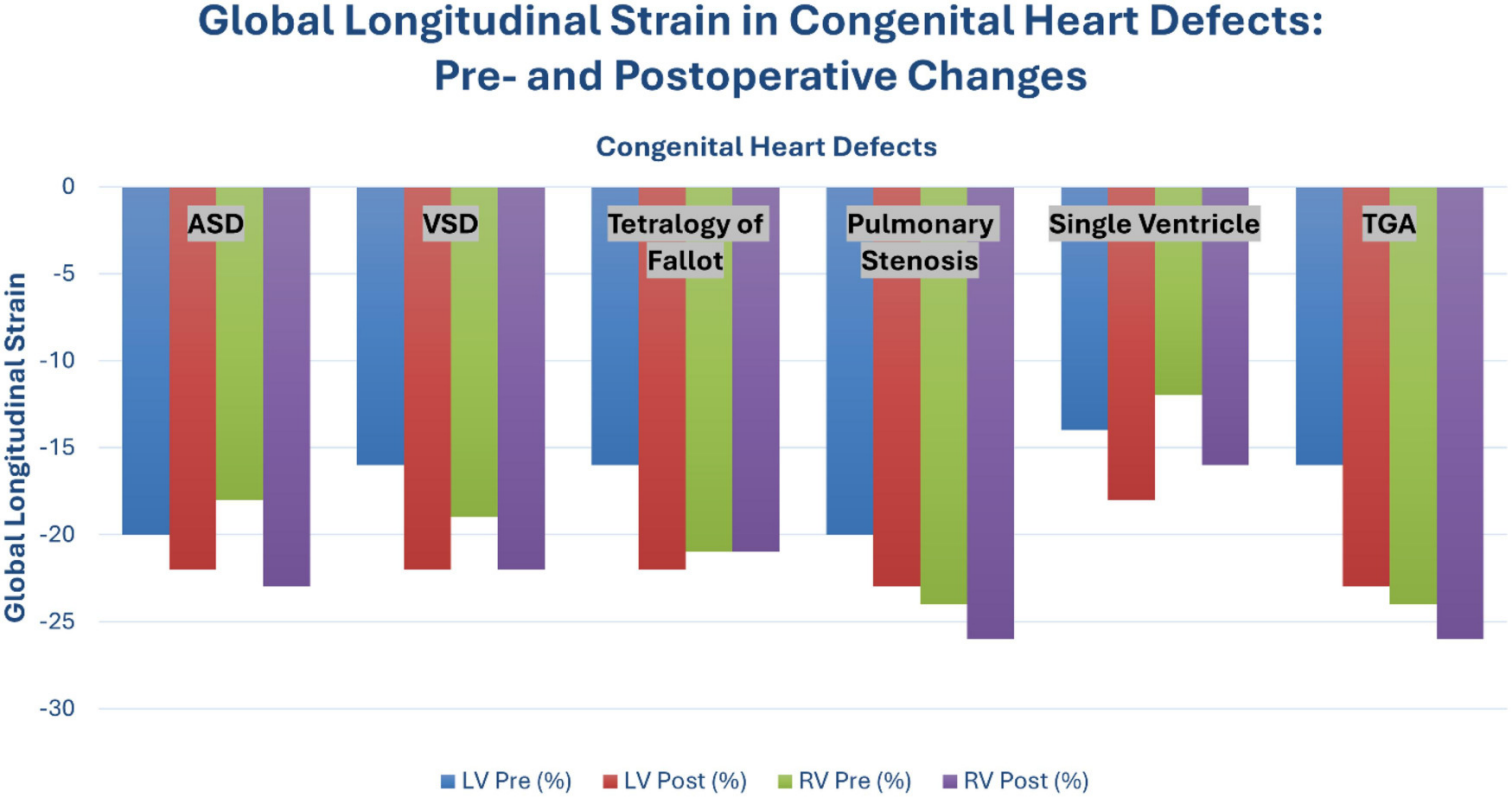
Global longitudinal strain (GLS) in selected congenital heart defects before and after surgical correction. Values represent average or median GLS from echocardiographic studies. A significant reduction in ventricular strain—particularly in the right ventricle (RV)—was observed postoperatively, highlighting its sensitivity as a marker of subclinical dysfunction.

Similarly, postoperative evaluations consistently demonstrate a significant reduction in longitudinal strain across both the right and left ventricles. As illustrated by the data in Figure 3, this biventricular functional decline is evidenced by a decrease in median LV strain from −20% to −15.6%, and RV strain from −17.8% to −15.63% by the time of hospital discharge. Notably, this reduction is more pronounced in patients exposed to prolonged cardiopulmonary bypass or aortic cross-clamp times. This suggests a direct correlation between the duration of extracorporeal support and the degree of perioperative myocardial injury (39, 40). Consequently, these early subclinical changes carry important prognostic implications for long-term clinical outcomes, particularly regarding the trajectory of persistent cardiac dysfunction.

## 6 Strain findings in specific congenital heart diseases

### 6.1 Atrial septal defect (ASD)

Patients with ASD may remain asymptomatic in the early stages but can later develop clinical manifestations such as dyspnea, palpitations, and irregular heartbeats. In advanced adulthood, chronic RV volume overload may lead to pulmonary hypertension, resulting in progressive RV dysfunction and, in severe cases, multiorgan failure (41).

Initially, an ASD causes volume overload before the rise in pulmonary vascular resistance leads to increased RV afterload. During follow-up, elevated RV longitudinal strain values have been observed prior to defect closure, which typically decreases after surgical correction. However, some strain elevation may persist due to ventricular remodeling and residual RV volume (12, 41).

About 10% of ASD patients progress to pulmonary arterial hypertension or precapillary pulmonary hypertension, driving RV remodeling with characteristic dilation and hypertrophy. These structural changes compromise RV function, manifesting as elevated longitudinal strain values (41).

In patients with ASD, while myocardial velocities remain preload-sensitive, RV volume overload elevates global strain values, which normalize post-correction. Strain rate, being less preload-dependent, serves as a more reliable marker of chronic preoperative ventricular adaptation (23).

While RV systolic parameters (inclu|ding strain) generally normalize post-closure, an initial decline is often observed. Notably, preserved free-wall longitudinal strain, concurrent with apical strain elevation, may represent regional compensation mechanisms (18).

In repaired ASD patients, GLS effectively predicts early ventricular dysfunction and guides the optimal timing of medical therapy for volume overload (42).

### 6.2 Ventricular septal defect (VSD)

In patients undergoing ventricular septal repair, abnormal LV- GLS (cutoff value ≤−20.1%) was associated with a higher incidence of adverse events (including sudden death, cardiac arrest, ECMO requirement, and hepatic/renal dysfunction), prolonged mechanical ventilation duration, and extended ICU and hospital stays. Thus, LV-GLS serves as a significant predictor of adverse outcomes following corrective surgery (43).

### 6.3 Aortic coarctation

Measurement of LV longitudinal strain during the fetal period has emerged as a valuable diagnostic tool for early detection of aortic coarctation. This technique not only identifies functional abnormalities before birth but also guides planning for postnatal surgical management. A cutoff value of −12.8% has been proposed, below which moderate-to-severe risk may justify surgical intervention at birth (44).

These findings reflect compensatory LV dysfunction, where combined afterload elevation and structural remodeling occur secondary to systemic outflow obstruction (44).

### 6.4 Tetralogy of Fallot

Tetralogy of Fallot (TOF) is frequently associated with chronic RV volume overload, contributing to progressive RV dysfunction. This functional impairment is linked to increased morbidity and mortality, primarily due to heart failure and potentially lethal ventricular arrhythmias—including sudden cardiac death in adulthood (7, 45).

In this context, early longitudinal assessment of RV function using strain imaging serves as a key tool for risk stratification, therapeutic planning, and prognostic optimization.

Following surgical correction of TOF, most patients develop pulmonary valve insufficiency, contributing to late-onset RV dysfunction. In this setting, GLS detects subclinical biventricular impairment (42). STE evaluates RV mechanics through multidirectional analysis (longitudinal, circumferential, and radial motion), with RV GLS emerging as the preferred parameter for assessing systolic function (7, 45).

The mean longitudinal strain and strain rate values in the RV free wall, particularly in its basal, mid, and apical segments, have been identified as predictors of cardiovascular events (7). Reduced longitudinal strain and strain rate reflect decreased myocardial contractility and impaired deformation, indicating RV dysfunction. These changes clinically manifest as diastolic ventricular dilation and ineffective systolic contraction, with a progressive increase in ventricular volume (7, 18, 46). Consequently, TOF patients exhibit fewer negative strain values in the context of RV failure compared to healthy controls (7, 45, 46).

Both LV-GLS and RV free-wall strain have been established as independent prognostic factors for mortality and heart failure hospitalization. Notably, reduced LV circumferential strain and RV longitudinal strain are associated with a higher risk of sudden cardiac death, need for cardiopulmonary resuscitation, or ventricular tachycardia, even when EF remains preserved (47, 48).

Following surgical correction, progressive improvement in RV longitudinal strain is observed, particularly after the age of two. However, these values remain below those observed in healthy controls, indicating that abnormal ventricular deformation patterns are present from birth and may persist in the long term (45, 47).

Concurrently, LV diastolic mechanics are typically impaired, demonstrating reduced early and late diastolic strain rates compared to controls (47). Regarding the surgical approach (staged vs. primary repair), while no significant differences exist in GLS, a mild reduction in median strain values has been reported (45).

Quantitative segmental assessment reveals systolic impairment (reduced strain and strain rate) across all RV free-wall segments (basal, mid, apical), coupled with abnormal early and late diastolic relaxation patterns (47, 48).

Atrial dilation represents a late marker of diastolic dysfunction and has been associated with adverse cardiovascular events and increased mortality. While atrial function evaluation using strain and strain rate provides valuable clinical information, even in the absence of overt dilation, these measures are not routinely performed in clinical practice. In TOF patients, these parameters may become abnormal during the early stages of the disease (27).

Following pulmonary valve replacement, atrial function shows no significant improvement, unlike other echocardiographic parameters that demonstrate RV functional recovery. These findings persist even in cases with associated atrial septal defects, suggesting that atrial dysfunction may become established early and remain fixed despite surgical correction (27).

Patients with residual pulmonary stenosis following TOF repair demonstrate an inverse relationship between stenosis severity and RV longitudinal strain, indicating functional impairment despite preserved ejection fraction. This finding underscores the clinical value of strain imaging as an early diagnostic tool for determining the optimal timing of pulmonary valve replacement (7, 12, 18).

### 6.5 Ebstein's anomaly

A qualitative assessment of RV function and measurement of RV-FWS reveal a limited correlation with RV ejection fraction (RVEF) when using conventional methods, particularly in echocardiographic studies. However, this relationship is more accurately demonstrated using advanced imaging techniques, such as cardiac magnetic resonance (CMR), which enables a precise evaluation of ventricular volume and function (18).

Following surgical correction, RV-FWS demonstrates a stronger correlation with CMR-derived RVEF, suggesting strain imaging serves as a valuable parameter for long-term functional monitoring in these patients (42).

Notably, patients undergoing the Cone procedure for tricuspid valve repair often demonstrate impaired RV systolic function despite favorable clinical status. This subclinical dysfunction necessitates rigorous evaluation using RV-FWS—assessed by advanced echocardiography or CMR- as part of long-term follow-up (49).

### 6.6 Single ventricle

Ventricular dysfunction is a well-documented complication in single ventricle patients following Fontan circulation, attributed to unfavorable hemodynamic loading conditions (volume and/or pressure overload) (34). In these patients, global and segmental strain values are typically lower compared to those of healthy controls. Specifically, impaired basal, mid, and apical strain is observed in the ventricular septum; reduced strain affects basal and mid segments of the *left-type* single ventricle-free wall, and the Strain rate remains unchanged (50, 51).

Patients undergoing palliative procedures (Fontan operation, Blalock-Taussig shunt, pulmonary artery banding, or Norwood protocol) are at risk of developing heart failure, which may manifest as ventricular dysfunction, pulmonary vascular insufficiency, low cardiac output syndrome, and elevated systemic venous pressures (5).

In single ventricle physiology, particularly in tricuspid atresia, distinct patterns of myocardial fiber orientation have been documented. These patterns correlate with the size of the hypoplastic RV, ventriculoarterial alignment, and the dimensions of the contralateral (systemic) ventricle. This contrasts sharply with healthy hearts, where superficial oblique fibers predominate (5).

Echocardiographic studies in single-ventricle patients reveal significantly larger end-systolic dimensions of the left-type single ventricle compared to biventricular controls or healthy subjects. These patients also demonstrate an increased sphericity index, reduced LVEF, and characteristic regional strain abnormalities, including decreased strain in the basal septal and mid-anteroseptal segments contrasted with increased strain in the apical septal and apical lateral segments. The circumferential deformation profile shows impaired mid-basal end-systolic circumferential deformation, reduced global circumferential strain, and altered rotational mechanics manifesting as decreased basal rotation with increased apical rotation, resulting in enhanced LV twist. However, specific parameters, including LV dimensions, end-systolic longitudinal deformation, GLS, and mid-apical circumferential strain, remain stable regardless of the specific cardiac defect type (5, 52, 53).

Patients with either right or LV morphology single ventricle physiology show no significant differences in GLS or strain rate following Fontan surgery. However, a comparative analysis reveals lower strain values in the basal free wall and basal, mid, and apical interventricular septum in RV morphology, in contrast to higher apical free wall strain values. These regional differences, while notable, do not reach statistical significance (51).

Regarding regional strain rate, RV morphology demonstrates reduced values, specifically in the basal interventricular septum and basal free wall, with preserved strain rate in all other myocardial segments (51).

### 6.7 Hypoplastic left heart syndrome (HLHS)

The presence of a morphologic RV as the systemic ventricle is associated with significant morbidity and mortality. This stems from the ventricle's inherent inability to adapt to long-term systemic hemodynamic demands, ultimately leading to progressive deterioration of both systolic and diastolic function (54).

Fetal echocardiographic measurements can predict the postnatal clinical course: a reduced RV short-axis diameter and tricuspid annular diameter are associated with the need for early Glenn anastomosis. An enlarged mid-RV short-axis diameter correlates with delayed Glenn anastomosis (>4 months of age) (55).

While RV circumferential dilation with preserved systolic function is an expected mid-gestation finding without mortality implications, abnormal RV strain has been detected as early as fetal life. This suggests ventricular dysfunction begins *in utero*, though its prognostic significance for postnatal outcomes requires further validation (55).

Close monitoring of ventricular function is crucial for patients with HLHS following Fontan completion. Early detection of ventricular dysfunction enables timely intervention and improves clinical outcomes. STE has proven particularly valuable for prognostic stratification, with global circumferential strain (GCS) emerging as an independent predictor of mortality and transplant-free survival and a parameter unaffected by ventricular geometry or Doppler angle limitations (23, 54).

In this physiology, where the RV assumes systemic function, patients demonstrate a higher prevalence of neoaortic insufficiency and tricuspid stenosis, which leads to volume overload, ventricular dilation, and progressive systolic deterioration (56). These hemodynamic alterations result in inadequate cardiac output, accompanied by elevated Fontan circuit pressures, which produce clinically significant consequences. Echocardiographic analysis reveals a characteristic pattern of predominant circumferential shortening over longitudinal deformation, reflecting the mechanical adaptation of the systemic RV to its new functional role (54, 55, 57).

Throughout the surgical staging for Fontan completion, progressive deterioration of longitudinal ventricular function has been consistently observed. During the pre-Norwood phase, patients demonstrate relative enlargement of RV diameter accompanied by reduced longitudinal strain, while circumferential deformation remains preserved (56). Following the Norwood procedure, impaired RV systolic function emerges as a significant risk factor for mortality or transplant requirements. This functional decline persists even after Fontan's completion, with documented reductions in GLS that correlate with long-term outcomes (54, 55).

In contrast to longitudinal strain, circumferential strain remains stable throughout all surgical stages. While longitudinal deformation serves as the more sensitive parameter for early prediction of adverse events prior to Stage II palliation, circumferential strain demonstrates superior prognostic utility in advanced stages. Following Fontan's completion, the circumferential strain shows a particularly strong correlation with mortality and transplant requirement, emerging as the more reliable indicator of long-term outcomes (54, 56, 58).

Regional assessment of the systemic RV reveals significantly reduced deformation in lateral segments (corresponding to the free wall), likely attributable to decreased preload. This pattern may reflect mechanical interference from fibrotic remnants of the LV, particularly along the septal region, though global RV strain appears preserved (23). Furthermore, acute afterload elevation produces measurable reductions in GLS rate without altering end-systolic elastance, demonstrating this parameter's sensitivity to loading conditions (23).

### 6.8 Transposition of the great arteries (TGA)

In patients with a morphological RV functioning as the systemic ventricle (e.g., following Mustard or Senning procedures), serial assessment of RV function is essential. Among available echocardiographic parameters, myocardial strain has gained particular prognostic relevance for detecting progressive dysfunction. RV longitudinal strain evaluation is specifically recommended due to its sensitivity in identifying subclinical systolic dysfunction (18).

In contrast to the pulmonary RV, the systemic RV develops mechanical properties more similar to those of the LV, demonstrating predominant circumferential shortening and absence of torsional deformation. This adaptation results in a relative reduction of longitudinal strain, which reflects the ventricle's characteristic longitudinal myocyte fiber orientation (12, 35, 59). Measurement of longitudinal shortening provides valuable complementary data for comprehensive assessment of systemic RV systolic function following atrial switch procedures (35).

Patients with systemic RVs demonstrate a characteristic strain pattern consisting of mildly reduced GLS and significantly diminished transverse strain, with relative preservation only in basal and mid-free-wall segments. In contrast, circumferential strain typically increases as a compensatory response to elevated afterload, representing a functional adaptation to maintain cardiac output (12, 18, 35). This relative reduction in longitudinal deformation has emerged as a prognostic marker for progressive systemic ventricular dysfunction or failure.

The LV geometry significantly influences systemic RV mechanics. Characteristic apical bulging of the RV has been documented, contributing to regional strain reduction in this area (18). Notably, basal free-wall segment strain may remain preserved or even increase as a compensatory mechanism to maintain cardiac output. In contrast, interventricular septal longitudinal strain typically demonstrates greater impairment compared to free-wall strain, reflecting biventricular interdependence as a key limiting factor for functional capacity and exercise tolerance (35).

Comparative studies demonstrate significantly reduced mean strain values in the interventricular septum (particularly mid and apical segments) of systemic RVs compared to healthy controls (60). However, fetal evaluations reveal preserved GLS with comparable values between both ventricles and normal fetuses, a finding attributable to fundamental differences in fetal circulatory physiology (59).

Over time, the systemic RV develops significant trabecular hypertrophy as an adaptive response to high-pressure circulation. However, this compensatory mechanism has inherent limitations, with progressive deterioration of systemic ventricular function ultimately leading to increased cardiovascular event risk and mortality (35).

Both strain and strain rate are inherently influenced by preload, though with important physiological distinctions: strain demonstrates greater sensitivity to acute preload variations, while strain rate remains relatively less affected by these dynamic changes. This differential preload dependence carries particular clinical relevance when interpreting systemic RV function, revealing fundamental differences from the non-systemic RV's mechanical behavior (23).

## 7 Strain analysis in pulmonary hypertension associated with cardiac diseases

Strain parameters hold significant prognostic value in pulmonary hypertension patients. Both RV free-wall strain and right atrial strain have been established as robust predictors of clinical outcomes in this population (18). In patients with pulmonary hypertension secondary to cardiac disease, diastolic strain rate serves as a useful tool for assessing RV diastolic function. However, this parameter has limitations in differentiating early from late diastolic abnormalities, as it cannot reliably distinguish between impaired myocardial relaxation and reduced ventricular compliance (28).

Patients with pulmonary hypertension demonstrate characteristic LV diastolic dysfunction, manifested by impaired myocardial deformation during early diastole. This pathophysiology is quantified through significantly reduced global basal circumferential diastolic strain rate, particularly in septal and lateral segments (61). A parallel reduction in global and mid-ventricular longitudinal diastolic strain rate is observed compared to healthy controls, indicating that LV myocardial dysfunction extends beyond conventional filling pattern abnormalities. These findings establish strain as a sensitive marker for early myocardial impairment (61, 62). Concurrently, these patients exhibit significantly diminished global and regional longitudinal strain in the RV free wall compared to controls (61).

The LV strain reduction predominantly localizes to the interventricular septum, primarily reflecting impaired longitudinal strain in the basal and mid-septal segments. These LV strain abnormalities show a significant correlation with pulmonary hypertension severity (61).

## 8 Strain analysis in congenital heart disease: fetal applications

Fetal echocardiography represents an essential diagnostic tool for the early detection of congenital cardiac malformations. This imaging modality improves postnatal prognosis by facilitating comprehensive differential diagnosis, enabling potential intrauterine interventions, guiding individualized delivery planning, and optimizing perinatal care strategies (1).

In this clinical context, fetal myocardial strain measurement serves as an indirect yet sensitive marker of cardiac function, providing valuable physiological data even in healthy fetuses (1). This advanced technique can detect subclinical functional abnormalities that conventional structural assessments fail to identify.

In fetuses without cardiac pathology, GLS and strain rate demonstrate a physiological progression, with values becoming arithmetically higher (less negative) with advancing gestational age (63, 64). Furthermore, RV strain consistently shows higher values (less negative) compared to LV strain, reflecting inherent physiological differences in fetal ventricular function (63).

Strain assessment has proven to be particularly valuable for identifying high-risk fetuses, specifically those who will be small for gestational age (SGA) due to placental insufficiency. In these cases, right ventricular systolic dysfunction has been documented, manifested by a significant reduction in deformation—indicated by increased (less negative) RV-FWS values. This alteration is evident before 23 weeks of gestation and precedes the onset of overt growth restriction. Therefore, these early changes in RV myocardial deformation serve as sensitive markers of fetal hemodynamic compromise, enabling the identification of at-risk fetuses who might go unnoticed with conventional biometry or who have not yet developed Doppler abnormalities (65).

**Figure 4:**
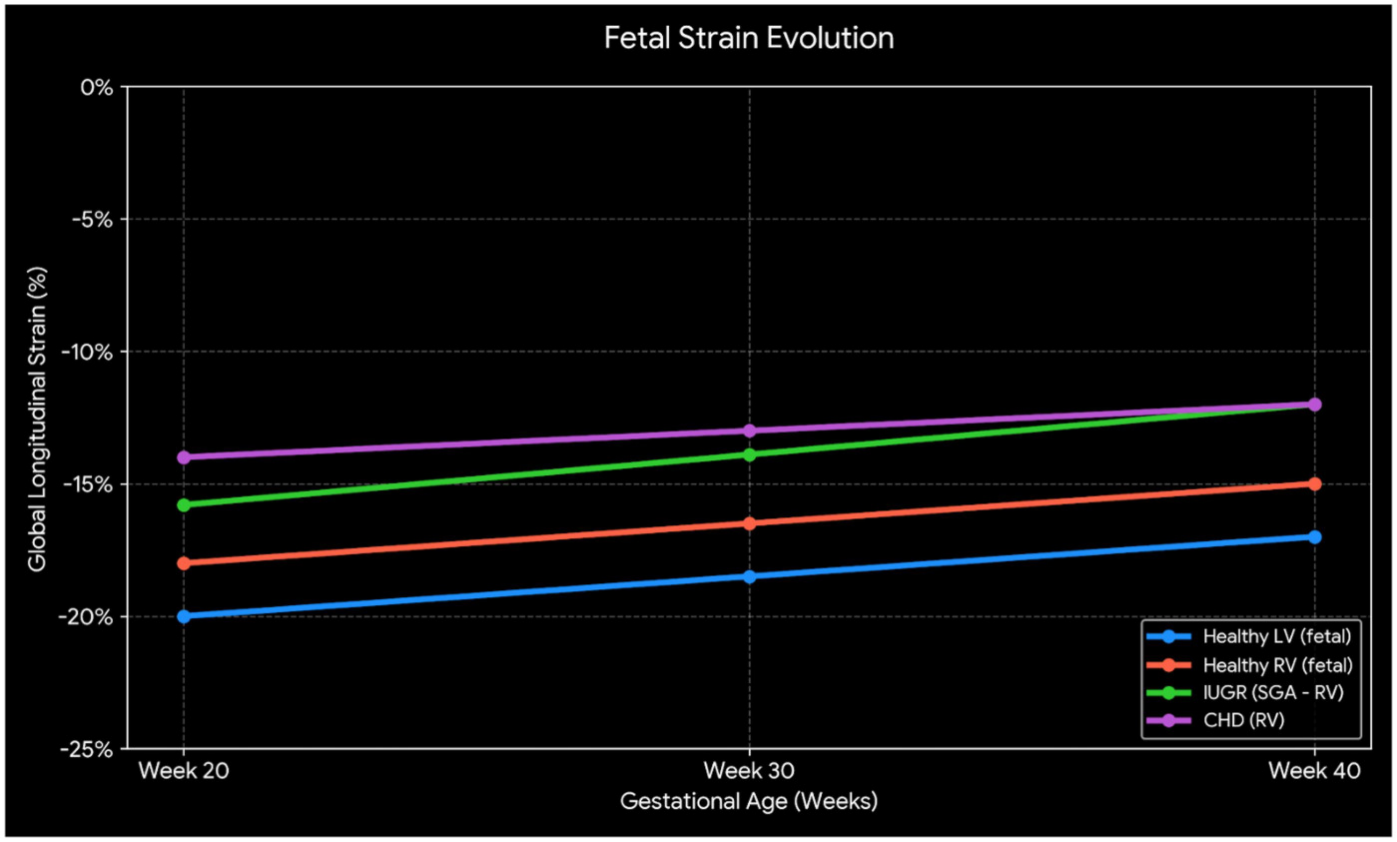
Evolution of fetal global longitudinal strain (GLS). Physiological and pathological trends across gestation. * Healthy LV/RV: Based on longitudinal data from van Oostrum et al. (63), showing values becoming progressively less negative (arithmetically increasing) toward term. * IUGR (SGA): Represents RV-GLS values. Based on van Oostrum et al. (65), these fetuses exhibit significantly less negative strain (systolic dysfunction) as early as week 23. * CHD: Represents RV-GLS values. Synthesis based on van den Wildenberg et al. (64), illustrating a persistent reduction in deformation (less negative values) compared to healthy controls throughout gestation.

Similarly, fetuses with CHD exhibit persistent subclinical dysfunction. Across common obstructive lesions, RV-FWS demonstrates a significant and persistent reduction in deformation (less negative values) compared to healthy controls throughout gestation (64). The divergence between physiological biventricular maturation and these pathological right-ventricular trajectories (such as in SGA/IUGR and CHD) is conceptually illustrated in Figure 4.

While these findings highlight the immense potential of STE for early risk stratification, routine clinical implementation should be approached with measured optimism. Current evidence suggests that strain imaging serves as a highly valuable adjunctive tool in specialized centers (64), but its broad systematic adoption awaits the development of definitive consensus guidelines.

Since ventricular dysfunction in the fetal heart typically manifests first in the RV, comprehensive evaluation of both ventricles is essential, accounting for their functional interdependence in global cardiac performance (1).

Fetal echocardiographic strain analysis faces several technical challenges, including the small absolute dimensions of the fetal heart, limited tracking area due to myocardial thinness, and motion artifacts from fetal movement. These factors may compromise image consistency and strain measurement precision (1).

## 9 Alternative strain imaging modalities

### 9.1 Cardiac magnetic resonance (CMR)

Several imaging modalities complement echocardiography for myocardial deformation assessment. Among these, CMR represents the gold standard for precise evaluation of ventricular and atrial function, volume, and mass (2–4). This technique provides high spatial and temporal resolution imaging with excellent reproducibility, overcoming inherent echocardiographic limitations such as inadequate acoustic windows or inconsistent visualization of all cardiac segments (4, 6).

CMR enables comprehensive myocardial strain quantification, with particular utility for RV assessment. This modality accurately reflects both systolic and diastolic ventricular function, proving especially valuable for early detection of subclinical cardiac mechanical dysfunction and longitudinal monitoring of cardiovascular disease progression prior to clinical manifestation (2).

### 9.2 Cardiac computed tomography (CCT)

CCT has emerged as a viable alternative for strain and atrial volume assessment, particularly in pediatric patients with limited acoustic windows or contraindications to CMR (3). This modality provides exceptional spatial and temporal resolution, making it valuable for postoperative evaluation of LV global strain in CHD patients where transthoracic echocardiography may be suboptimal (6).

The primary drawback of CCT remains ionizing radiation exposure, particularly concerning pediatric populations. Additionally, technical constraints exist, including inherent difficulties in assessing global radial strain. This limitation stems from CCT's reliance on endocardial tracking, which fails to adequately capture deformation patterns across the full myocardial wall thickness (6).

## 10 Conclusions

Myocardial strain analysis, particularly through speckle-tracking echocardiography, has emerged as a highly valuable tool for the functional cardiac assessment of pediatric and fetal patients with CHD. Unlike traditional parameters such as ejection fraction, strain imaging provides enhanced sensitivity for detecting subclinical mechanical dysfunction at both ventricular and atrial levels. The technique's capacity for multidimensional assessment—evaluating longitudinal, circumferential, and radial planes—proves highly advantageous for characterizing ventricular function in complex anatomies and monitoring chronic hemodynamic overload.

Current observational evidence highlights the clinical utility of myocardial strain across three critical domains: early detection of ventricular dysfunction, postoperative surveillance, and risk stratification. Of particular significance, RV-FWS has shown promise as a sensitive marker of functional impairment in conditions such as Tetralogy of Fallot, single ventricle physiology, and transposition of the great arteries. Similarly, fetal strain analysis has emerged as a useful adjunct to conventional structural echocardiography, aiding in the early detection of incipient dysfunction and facilitating personalized perinatal management.

However, the interpretation of these parameters in the pediatric population requires a highly nuanced, expert approach. Strain values must be contextualized rather than treated as absolute, universal cut-offs. Normative ranges exhibit significant variability related to age, body surface area, and proprietary vendor software differences (21, 26). Furthermore, clinicians must carefully distinguish between load-dependent myocardial deformation and true intrinsic contractility, particularly given the complex, altered hemodynamic loading conditions characteristic of CHD (26).

In summary, while echocardiographic strain analysis significantly enhances traditional cardiac assessment, its systematic clinical implementation should be approached with measured optimism. Rather than being positioned as a fundamentally necessary replacement for current standards, it currently serves as a powerful adjunctive tool in specialized pediatric CHD centers. The transition of strain imaging from an advanced supplementary technique to a definitive, standalone guide for clinical decision-making will require the establishment of robust, age-stratified, vendor-neutral pediatric reference ranges, alongside prospective multicenter trials to validate its direct impact on long-term clinical outcomes.

## References

[1] Drop MCV Möllers M Hammer K Oelmeier De Murcia K Falkenberg MK Braun J Strain and dyssynchrony in fetuses with congenital heart disease compared to normal controls using speckle tracking echocardiography (STE). J Perinat Med. (2019) 47:598–604. 10.1515/jpm-2019-0073 31141490

[2] Li Z Liang Y Cheng S Xie B Zhang S Liu X Evaluation of RV myocardial strain in pulmonary arterial hypertension associated with atrial septal defect by cardiac magnetic resonance feature tracking. Int J Cardiovasc Imaging. (2022) 38:2035–45. 10.1007/s10554-022-02591-2 37726610

[3] Xie WH Chen LJ Hu LW Ouyang RZ Guo C Sun AM Cardiac computed tomography-derived left atrial strain and volume in pediatric patients with congenital heart disease: a comparative analysis with transthoracic echocardiography. Front Cardiovasc Med. (2022) 9:870014. 10.3389/fcvm.2022.870014 35795359 PMC9251122

[4] Voges I Negwer I Caliebe A Boroni Grazioli S Daubeney PEF Uebing A Myocardial deformation in the pediatric age group: normal values for strain and strain rate using 2D magnetic resonance feature tracking. J Magn Reson Imaging. (2022) 56:1382–92. 10.1002/jmri.28073 35072310

[5] Lopez C Mertens L Dragulescu A Landeck B Younoszai A Friedberg MK Strain and rotational mechanics in children with single LVs after Fontan. J Am Soc Echocardiogr. (2018) 31:1297–306. 10.1016/j.echo.2018.09.004 30344011

[6] Xie WH Chen LJ Hu LW Ouyang RZ Guo C Sun AM Postoperative evaluation of LVglobal strain using cardiac computed tomography in pediatric patients with congenital heart disease: a comparison with echocardiography. Eur J Radiol. (2021) 142:2, 5–7. 10.1016/j.ejrad.2021.109868 34311155

[7] Kamal NM Salih AF Ali BM. Speckle tracking echocardiography for diagnosis of RV failure in children with totally corrected tetralogy of Fallot in Sulaimani, Iraq. J Taibah Univ Med Sci. (2024) 19:198–208. 10.1016/j.jtumed.2023.11.005 38124989 PMC10730916

[8] Abou R Van Der Bijl P Bax JJ Delgado V. Global longitudinal strain: clinical use and prognostic implications in contemporary practice. Heart. (2020) 106(18):1438–44. 10.1136/heartjnl-2019-316215 32404401

[9] Melo S Alzate-Ricaurte S Pedroza S Moreno M Largo J Rivera R Optimal global longitudinal strain thresholds for pediatric heart surgery: insights from a university hospital. Pediatr Cardiol. (2024) 45:780–6. 10.1007/s00246-024-03437-5 38421480

[10] Domenech RJ Parra VM. Contractilidad ventricular: Fisiología y proyección clínica. Rev Med Chil. (2016) 144(6):767–71. 10.4067/S0034-98872016000600012 27598497

[11] Zarco P. Estructura cardíaca, mecánica de la contracción y miocardiopatías dilatadas contracción miocárdica/fisiopatología/miocardiopatía congestiva (1998). 10.1016/s0300-8932(98)74785-2 9711099

[12] Muraru D Haugaa K Donal E Stankovic I Voigt JU Petersen SE RV Longitudinal strain in the clinical routine: a state-of-the-art review. Eur Heart J Cardiovasc Imaging (2022) 23:898–912. 10.1093/ehjci/jeac022 35147667

[13] Land S Niederer SA. Influence of atrial contraction dynamics on cardiac function. Int J Numer Method Biomed Eng. (2018) 34(3):e2931. 10.1002/cnm.2931 28990354

[14] Padala SK Cabrera JA Ellenbogen KA. Anatomy of the cardiac conduction system. Pacing Clin Electrophysiol. (2021) 44(1):15–25. 10.1111/pace.14107 33118629

[15] Heidenreich PA Bozkurt B Aguilar D Allen LA Byun JJ Colvin MM AHA/ACC/HFSA guideline for the management of heart failure: executive summary: a report of the American College of Cardiology/American Heart Association joint committee on clinical practice guidelines. Circulation. (2022) 145(18):E876–94. 10.1161/CIR.0000000000001062 35363500

[16] Rudski LG Lai WW Afilalo J Hua L Handschumacher MD Chandrasekaran K Guidelines for the echocardiographic assessment of the right heart in adults: a report from the American society of echocardiography. J Am Soc Echocardiogr. (2010) 23(7):685–713. 10.1016/j.echo.2010.05.010 20620859

[17] Lu KJ Chen JXC Profitis K Kearney LG Desilva D Smith G RV global longitudinal strain is an independent predictor of RV function: a multimodality study of cardiac magnetic resonance imaging, real-time three-dimensional echocardiography, and speckle tracking echocardiography. Echocardiography. (2015) 32:966–74. 10.1111/echo.12783 25287078

[18] Mah K Mertens L. Echocardiographic assessment of RV function in paediatric heart disease: a practical clinical approach. CJC Pediatr Congenit Heart Dis. (2022) 1:136–57. 10.1016/j.cjcpc.2022.05.002 37970496 PMC10642122

[19] Neuser J Buck HJ Oldhafer M Sieweke JT Bavendiek U Bauersachs J RV function improves early after percutaneous mitral valve repair in patients suffering from severe mitral regurgitation. Front Cardiovasc Med. (2022) 9:830944. 10.3389/fcvm.2022.830944 35369337 PMC8968125

[20] Abraham TP Dimaano VL Liang HY. Role of tissue Doppler and strain echocardiography in current clinical practice. Circulation. (2007) 116(22):2597–609. 10.1161/CIRCULATIONAHA.106.647172 18040039

[21] Romanowicz J Ferraro AM Harrington JK Sleeper LA Adar A Levy PT Pediatric normal values and Z score equations for left and right ventricular strain by two-dimensional speckle-tracking echocardiography derived from a large cohort of healthy children. J Am Soc Echocardiogr. (2023) 36(3):310–23. 10.1016/j.echo.2022.11.006 36414123

[22] Levy PT Machefsky A Sanchez AA Patel MD Rogal S Fowler S Reference ranges of LVStrain measures by two-dimensional speckle-tracking echocardiography in children: a systematic review and meta-analysis. J Am Soc Echocardiogr. (2016) 29:209–25.e6. 10.1016/j.echo.2015.11.016 26747685 PMC4779733

[23] Schlangen J Petko C Hansen JH Michel M Hart C Uebing A Two-dimensional global longitudinal strain rate is a preload-independent index of systemic RV contractility in hypoplastic left heart syndrome patients after Fontan operation. Circ Cardiovasc Imaging. (2014) 7:880–6. 10.1161/CIRCIMAGING.114.002110 25270741

[24] Boczar KE Sarwar S Hakimjavadi R Abumustafa Y Kadoya Y Paterson DI. Multimodality imaging to understand mechanisms of RV disease. Can J Cardiol. (2025) 11(2P1):260–74. 10.1016/j.cjca.2025.03.033 40188873

[25] Anastasiou V Papazoglou AS Moysidis DV Daios S Tsalikakis D Giannakoulas G The prognostic value of RV longitudinal strain in heart failure: a systematic review and meta-analysis. Heart Fail Rev. (2023) 28(6):1383. 10.1007/s10741-023-10329-y 37308615 PMC10575809

[26] Thomas JD Edvardsen T Abraham T Appadurai V Badano L Banchs J Clinical applications of strain echocardiography: a clinical consensus statement from the American society of echocardiography developed in collaboration with the European association of cardiovascular imaging of the European Society of Cardiology. J Am Soc Echocardiogr. (2025) 38:1003–4, 1008. 10.1016/j.echo.2025.07.007 40864001

[27] Ittleman BR Tretter JT Bader AS Mcollum S Shabanova V Steele JM. Longitudinal evaluation of atrial function in patients with tetralogy of Fallot. Pediatr Cardiol. (2024) 46:835, 836, 840. 10.1007/s00246-024-03503-y 38849600

[28] Levy PT Clinical applications of strain in pediatric echocardiography. JACC Cardiovasc Imaging. (2020) 13(3):850–66.

[29] Friedberg MK Mertens L. Myocardial mechanics in congenital heart disease. Circ Cardiovasc Imaging. (2021).34875842

[30] Patel S Husain N Acevedo J Samples S Hauck A. A pathway for improving performance and interpretation of strain in a pediatric echocardiography laboratory. Echocardiography. (2025) 42(12):e70369. 10.1111/echo.70369 41383062 PMC12699170

[31] Gozar L Sasaran MO Toma D Cerghit-Paler A Molnar-Varlam C Mărginean C Left ventricular function evaluation of the fetal heart: reference intervals and inter-observer variability of 2D speckle-tracking echocardiography measurements. Med Ultrason. (2023) 25(2):168–74. 10.11152/mu-4021 37369048

[32] Joosen RS Meulblok EAM Mauritz-Fuite EH Slieker MG Breur JMPJ. Right ventricular strain in healthy children: Insights from Speckle-Tracking Echocardiography. J Cardiovasc Dev Dis. (2025) 12(9):322. 10.3390/jcdd12090322 41002601 PMC12470735

[33] Kühle H Cho SK Barber N Goolaub DS Darby JR Morrison JL Advanced imaging of fetal cardiac function. Front Cardiovasc Med. (2023) 10:1206138. 10.3389/fcvm.2023.1206138 37288263 PMC10242056

[34] Toro KD Soriano BD Buddhe S. RV global longitudinal strain in repaired tetralogy of Fallot. Echocardiography. (2016) 33:1557–62. 10.1111/echo.13302 27543374

[35] Okumura K Slorach C Mroczek D Dragulescu A Mertens L Redington AN RV diastolic performance in children with pulmonary arterial hypertension associated with congenital heart disease: correlation of echocardiographic parameters with invasive reference standards by high-fidelity micromanometer catheter. Circ Cardiovasc Imaging. (2014) 7:491–501. 10.1161/CIRCIMAGING.113.001071 24577356

[36] Ladouceur M Redheuil A Soulat G Delclaux C Azizi M Patel M Longitudinal strain of systemic RV correlates with exercise capacity in adult with transposition of the great arteries after atrial switch. Int J Cardiol. (2016) 217:28–34. 10.1016/j.ijcard.2016.04.166 27179205

[37] Pascual ES Zurita MB Sebastián JD Silva LGG Peinado AA Aguado FGL. Comparison of myocardial deformation by speckle-tracking echocardiography and cardiac magnetic resonance in patients with Fontan circulation: diagnostic algorithm. J Cardiovasc Echogr (2021) 31:144–50. 10.4103/jcecho.jcecho_126_20 34900549 PMC8603771

[38] Hassan MA Al-Akhfash A Bhat Y Alqwaiee A Abdulrashed M Almarshud SS Myocardial deformation in children post cardiac surgery, a cross-sectional prospective study. Egypt Heart J. (2024) 76:151. 10.1186/s43044-024-00578-z 39556306 PMC11573953

[39] Gozar L Iancu M Gozar H Sglimbea A Cerghit Paler A Gabor-Miklosi D Assessment of biventricular myocardial function with 2-dimensional strain and conventional echocardiographic parameters: a comparative analysis in healthy infants and patients with severe and critical pulmonary stenosis. J Pers Med. (2022) 12:57. 10.3390/jpm12010057 35055372 PMC8780169

[40] de Boer JM Kuipers IM Klitsie LM Blom NA ten Harkel ADJ. Decreased biventricular longitudinal strain shortly after congenital heart defect surgery. Echocardiography. (2017) 34:446–52. 10.1111/echo.13456 28168740

[41] Klitsie LM Roest AAW Blom NA Ten Harkel ADJ. Ventricular performance after surgery for a congenital heart defect as assessed using advanced echocardiography: from Doppler flow to 3d echocardiography and speckle-tracking strain imaging. Pediatr Cardiol. (2014) 35:3–15. 10.1007/s00246-013-0802-5 24121730

[42] Rahmianti ND Dinarti LK Mumpuni H Triastuti F. Global longitudinal strain RV (GLS RV) as a predictor for mean pulmonary artery pressure (MPAP) on secundum atrial septal defect (ASD) with pulmonary hypertension. J Cardiovasc Echogr. (2023) 33:83–7. 10.4103/jcecho.jcecho_14_23 37772046 PMC10529289

[43] Oliveira ALA de Oliveira MEP Guimarães LV Trindade GM Chaves GM Gonçalves ACP Evaluation of RV systolic function after tetralogy of Fallot repair: a systematic review comparing cardiac magnetic resonance and global longitudinal strain. Echocardiography. (2023) 40:4–14. 10.1111/echo.15486 36478414

[44] Ye H Ling Y An Q. Prognostic value of LVglobal longitudinal strain(LVGLS) in child patients undergoing the operation of ventricular septal defect (VSD). Asian J Surg. (2023) 46:5046–7. 10.1016/j.asjsur.2023.06.059 37419824

[45] Phillips AA Punn R Algaze C Blumenfeld YJ Chock VY Kwiatkowski DM LVStrain, arch angulation, and velocity-time integral ratio improve performance of a clinical pathway for fetal diagnosis of neonatal coarctation of the aorta. Fetal Diagn Ther. (2024) 51:320–34. 10.1159/000538550 38621375 PMC11318582

[46] Keelan J Pasumarti N Crook S Decost G Wang Y Crystal MA RV strain in patients with ductal-dependent tetralogy of Fallot. J Am Soc Echocardiogr. (2023) 36:654–65. 10.1016/j.echo.2023.03.006 36933850 PMC10281045

[47] Li VW Yu CK So EK Wong WH Cheung YF. Ventricular myocardial deformation imaging of patients with repaired tetralogy of Fallot. J Am Soc Echocardiogr. (2020) 33:788–801. 10.1016/j.echo.2020.03.017 32624088

[48] Zachos P Nevras V Milaras N Karakosta M Kalesi A Kasinos N The value of myocardial strain imaging in the evaluation of patients with repaired tetralogy of Fallot: a review of the literature. Heart Fail Rev. (2023) 28:97–112. 10.1007/s10741-022-10223-z 35286572

[49] Lianza AC Rodrigues ACT Mercer-Rosa L Vieira MLC de Oliveira WAA Afonso TR RV systolic function after the cone procedure for Ebstein’s Anomaly: comparison between echocardiography and cardiac magnetic resonance. Pediatr Cardiol. (2020) 41:985–95. 10.1007/s00246-020-02347-6 32335735

[50] Moiduddin N Texter KM Zaidi AN Hershenson JA Stefaniak C Hayes J Two-dimensional speckle strain and dyssynchrony in single LVs vs. normal LVs. Congenit Heart Dis. (2010) 5:579–86. 10.1111/j.1747-0803.2010.00460.x 21106018

[51] Petko C Hansen JH Scheewe J Rickers C Kramer HH. Comparison of longitudinal myocardial deformation and dyssynchrony in children with left and RV morphology after the Fontan operation using two-dimensional speckle tracking. Congenit Heart Dis. (2012) 7:16–23. 10.1111/j.1747-0803.2011.00607.x 22176662

[52] Strodka F Logoteta J Schuwerk R Salehi Ravesh M Gabbert DD Uebing AS Myocardial deformation in patients with a single LV using 2D cardiovascular magnetic resonance feature tracking: a case-control study. Int J Cardiovasc Imaging. (2021) 37:2549–59. 10.1007/s10554-021-02230-2 33788063 PMC8302517

[53] Hu L Sun A Guo C Ouyang R Wang Q Yao X Assessment of global and regional strain LVin patients with preserved ejection fraction after Fontan operation using a tissue tracking technique. Int J Cardiovasc Imaging. (2019) 35:153–60. 10.1007/s10554-018-1440-z 30121757

[54] Abdulkarim M Loomba RS Zaidi SJ Li Y Wilson M Roberson D Echocardiographic strain to predict need for transplant or mortality in Fontan patients with hypoplastic left heart syndrome. Pediatr Cardiol. (2024) 45:1475–84. 10.1007/s00246-023-03187-w 37204486

[55] Ittleman B Lowenstein S Edwards LA Caris E Bhat A Conwell J Fetal echocardiographic evaluation of tricuspid valve and RV function including global longitudinal strain in hypoplastic left heart syndrome and association with postnatal outcomes. Pediatr Cardiol. (2024) 46:1–3, 6–7. 10.1007/s00246-024-03453-5 38647657

[56] Menon SC Minich LL Casper TC Puchalski MD Hawkins JA Tani LY. Regional myocardial dysfunction following Norwood with RV to pulmonary artery conduit in patients with hypoplastic left heart syndrome. J Am Soc Echocardiogr. (2011) 24:826–33. 10.1016/j.echo.2011.05.008 21680148

[57] Balasubramanian S Smith SN Srinivasan P Tacy TA Hanley FL Chen S Longitudinal assessment of right ventricular function in hypoplastic left heart syndrome. Pediatr Cardiol. (2021) 42:1394–404. 10.1007/s00246-021-02624-y 33987707

[58] Petko C Voges I Schlangen J Scheewe J Kramer HH Uebing AS. Comparison of RV deformation and dyssynchrony in patients with different subtypes of hypoplastic left heart syndrome after Fontan surgery using two-dimensional speckle tracking. Cardiol Young. (2011) 21:677–83. 10.1017/S1047951111000631 21733343

[59] Young K Hooton C Zimmerman MB Reinking B Gupta U. Fetal left and RV strain parameters using speckle tracking in congenital heart diseases. Int J Cardiovasc Imaging. (2024) 40:1235–43. 10.1007/s10554-024-03094-y 38613605

[60] Toma D Gabor-Miklosi D Cerghit-Paler A Șuteu CC Cosma MC Mărginean C Impaired speckle-tracking-derived LVLongitudinal strain is associated with transposition of great arteries in neonates: a single-center study. Int J Environ Res Public Health. (2023) 20:8–9. 10.3390/ijerph20010674 PMC9820037 36612992

[61] Burkett DA Slorach C Patel SS Redington AN Ivy DD Mertens L LVmyocardial function in children with pulmonary hypertension: relation to RV performance and hemodynamics. Circ Cardiovasc Imaging. (2015) 8. 10.1161/CIRCIMAGING.115.003260 26259580 PMC4535191

[62] Burkett DA Slorach C Patel SS Redington AN Ivy DD Mertens L Impact of pulmonary hemodynamics and ventricular interdependence on LVDiastolic function in children with pulmonary hypertension. Circ Cardiovasc Imaging. (2016) 9:655, 657. 10.1161/CIRCIMAGING.116.004612 PMC5012318 27581953

[63] van Oostrum NHM de Vet CM Clur SB van der Woude DAA van den Heuvel ER Oei SG Fetal myocardial deformation measured with two-dimensional speckle-tracking echocardiography: longitudinal prospective cohort study of 124 healthy fetuses. Ultrasound Obstet Gynecol. (2022) 59:651–9. 10.1002/uog.24781 34558747 PMC9321172

[64] van den Wildenberg S van Beynum IM Havermans MEC Boersma E DeVore GR Simpson JM Fetal speckle tracking echocardiography measured global longitudinal strain and strain rate in congenital heart disease: a systematic review and meta-analysis. Prenat Diagn. (2024) 44:1493. 10.1002/pd.6672 39367541

[65] van Oostrum NHM van der Woude DAA Clur SAB Oei SG van Laar JOEH. Right ventricular dysfunction identified by abnormal strain values precedes evident growth restriction in small for gestational age fetuses. Prenat Diagn. (2020) 40(12):1525–31. 10.1002/pd.5805 32735353

