# Supplementary material for: Echocardiographic strain as an ally in the evaluation of congenital heart disease: a narrative review
Source: Front Cardiovasc Med. 2026 Apr 22;13:1662430. doi: 10.3389/fcvm.2026.1662430 (PMC13143851; doi:10.3389/fcvm.2026.1662430)
Supplement: Supplementary file 1 [file Table1.pdf]

Supplementary Data

1.1.Supplementary Tables

Cardiac Condition	Strain Abnormalities and Reference		
	**Clinical Context**	**Values**	**Clinical Implication**
**Atrial Septal Defect (ASD)**	Initially asymptomatic, later dyspnea and palpitations. Right ventricular (RV) volume overload may lead to pulmonary hypertension.	Elevated RV longitudinal strain before closure. Decreases after correction but may persist. Strain rate less preload-dependent.	Strain helps detect subclinical dysfunction and guide optimal timing for surgical repair.
**Ventricular Septal Defect (VSD)**	Associated with adverse events after surgery.	Left ventricular (LV) GLS <-20.1% predicts complications such as cardiac arrest, ECMO, and multiorgan dysfunction.	LV strain is useful for predicting postoperative complications and prolonged hospital stay.
**Aortic Coarctation**	Fetal LV dysfunction due to systemic outflow obstruction.	Fetal LV longitudinal strain < -12.8% indicates moderate to severe risk.	Allows early diagnosis and planning for postnatal surgical management.
**Tetralogy of Fallot (TOF)**	Chronic RV volume overload leads to progressive dysfunction and arrhythmias.	RV strain decreases after repair but remains below normal. Atrial strain also altered.	RV GLS predicts sudden cardiac death and heart failure, even with preserved ejection fraction.
**Ebstein´s Anomaly**	Subclinical RV dysfunction after Cone procedure despite good clinical condition.	Abnormal RV GLS; limited correlation with EF but strong correlation with MRI-derived RVEF.	RV strain recommended for long-term functional follow-up.
**Single Ventricle (Fontan)**	Unfavorable hemodynamics post-Fontan increase risk of dysfunction.	Reduced global and segmental strain. Circumferential strain better prognostic marker than longitudinal strain.	Strain imaging enables early detection and guides management in advanced stages.
**Hypoplastic Left Heart Syndrome (HLHS)**	Systemic right ventricle has poor long-term adaptation and deteriorates over time.	Reduced RV GLS from fetal life. Increased GCS post-Fontan correlates with survival.	GCS is a strong predictor of outcome after Fontan completion.
**Transposition of Great Arteries (TGA)**	Systemic RV develops maladaptive mechanics post-Mustard/Senning.	Decreased longitudinal strain, increased circumferential strain as compensation. Septal strain most affected.	Longitudinal strain is a predictor of systemic RV functional deterioration.
**Pulmonary Hypertension (Cardiac)**	RV overload and atrial dysfunction. Septal interaction affects LV function.	Decreased RV free-wall and RA strain. Reduced LV longitudinal and circumferential strain, especially in septal regions.	Strain detects early myocardial impairment and correlates with PH severity.

Supplementary Table 1: Strain Abnormalities and Reference. Overview of myocardial strain abnormalities across selected congenital and acquired cardiac conditions. The table summarizes the clinical context, key strain-derived parameters, and their clinical implications in each condition. Strain imaging—particularly global longitudinal strain (GLS)—provides valuable insights for early detection of subclinical myocardial dysfunction, prognostication, and surgical decision-making. Specific strain patterns vary by defect and can guide individualized patient monitoring and management strategies.
